# Lichen Planus

**DOI:** 10.3389/fmed.2021.737813

**Published:** 2021-11-01

**Authors:** Katharina Boch, Ewan A. Langan, Khalaf Kridin, Detlef Zillikens, Ralf J. Ludwig, Katja Bieber

**Affiliations:** ^1^Department of Dermatology, University of Lübeck, Lübeck, Germany; ^2^Dermatological Sciences, University of Manchester, Manchester, United Kingdom; ^3^Lübeck Institute of Experimental Dermatology, University of Lübeck, Lübeck, Germany; ^4^Azrieli Faculty of Medicine, Bar-Ilan University, Safed, Israel

**Keywords:** lichen planus, skin disease, inflammation, T-cell mediated, treatment

## Abstract

Lichen planus (LP) is a T cell-mediated disease affecting the stratified squamous epithelia of the skin and/or mucus membrane. Histologically, the disease is characterized by a lichenoid inflammatory infiltrate and vacuolar degeneration of the basal layer of the epidermis. LP has three major subtypes: Cutaneous, mucosal and appendageal LP. Rarely, it may affect the nails in the absence of skin and/or mucosal changes. LP may also be induced by several drugs, typically anti-hypertensive medication or be associated with infections, particularly viral hepatitis. The diagnosis is based on the clinical presentation and characteristic histological findings. Although the disease is often self-limiting, the intractable pruritus and painful mucosal erosions result in significant morbidity. The current first-line treatment are topical and/or systemic corticosteroids. In addition, immunosuppressants may be used as corticosteroid-sparing agents. These, however are often not sufficient to control disease. Janus kinase inhibitors and biologics (anti-IL-12/23, anti-IL17) have emerged as novel future treatment options. Thus, one may expect a dramatic change of the treatment landscape of LP in the near future.

## Introduction

**Figure 1:**
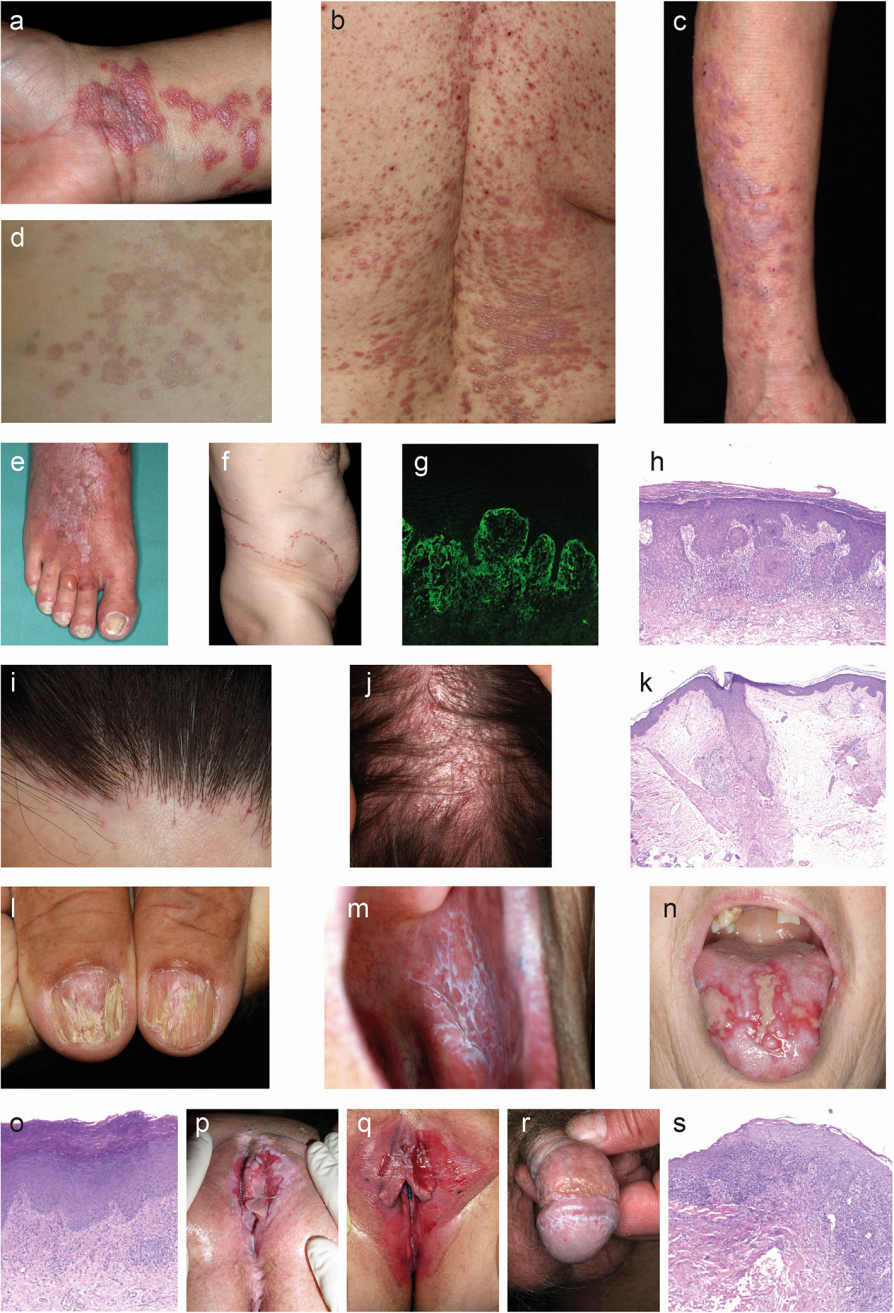
Clinical and histological hallmarks of lichen planus. **(a–f)** Cutaneous lichen planus (LP). **(a)** Polygonal, flat-topped, violaceous confuting plaques with fine white scales on the inner wrist in a patient with localized LP. **(b)** Symmetric red plaques on the back of a patient with generalized cutaneous LP. **(c)** Thick reddish-brown plaques on the arms of a patient with hypertrophic LP. **(e)** Blister on the 3rd toe along with widespread red plaques with whit streaks in a patient with lichen planus pemphigoides. **(f)** Linear lichen planus. **(g)** Direct immunofluorescence microcopy staining with fibrin deposition in the epidermis (400 ×). **(h)** The histology from a skin biopsy from a lichen planus lesion characteristically shows an irregularly epidermis with saw-toothed rete ridges, hypergranulosis, liquefaction degeneration of the dermal-epidermal junction and a lichenoid (band-like) lymphocytic infiltrate (H&E staining, 200 ×). **(i–l)** Appendageal LP. **(i)** Scaring alopecia and inflammation around hair follicles along the frontal scalp hair margin in a patient with frontal fibrosing alopecia. **(j)** Image from a patent with lichen planopilaris. **(k)** Lichenoid interface dermatitis of the hair infundibulum and apoptotic keratinocytes (Civatte bodies) and fibrous tracts in a biopsy from a patient with frontal fibrosing alopecia. **(l)** Grooved and ridged nails in a patient with nail LP. **(m–s)** Mucosal LP. **(m)** Wickham striae in the oral mucosa of a patient with oral LP. **(n)** Severe ulcera of the tounge in a patient with erosive oral LP. **(o)** Parakeratosis, acanthosis. band of inflammatory cells just beneath the epidermis, plasma cells in infiltrate in an oral biopsy from a patient with oral LP. **(p)** Severe vulval ulcerations in a patient with vulval LP. **(q)** Erythema and erosions in a patient with vulval LP. **(r)** Wickham striae on the glans penis in a patient with penile LP. **(s)** Occasional parakeratosis, irregularly thickened epidermis, apoptotic basal keratinocytes, lymphohistiocytic infiltrate in a biopsy from a patient with genital LP.

The term lichen planus (LP) stems from the Greek word “*leichen*,” which means “tree moss,” and the Latin word “*planus*,” which means “flat,” which aptly describes the surface of the cutaneous lesion (1). LP is a group of chronic inflammatory diseases affecting stratified squamous epithelia. Recently, LP is perceived as a T cell-mediated autoimmune disease, in which cytotoxic CD8+ T-cells are recruited into the skin and subsequently lead to an interface dermatitis (2–8). Viruses, drugs and contact allergens have all been reported to be possibly associated with development of LP (9–19). Clinically, LP is hallmarked by characteristic lesions, affecting the skin, hair, nails and/or mucous membranes. The classical skin changes are pruritic, purple, polygonal, flat-topped (planar) papules crossed by fine white lines, while erosions are seen on the mucous membranes (Figure 1). The latter may be associated with pain and/oral burning sensation (1). An overview of clinical subtypes and rare variants are listed in Table 1. LP preferentially affects middle-aged adults, with no known gender pre-disposition (1, 14). Whilst the clinical features are relatively characteristic, histological confirmation of the diagnosis is recommended to exclude potential differential diagnoses. The typical band-like lymphocytic infiltrate and interface dermatitis are the characteristic findings—irrespective of skin location or disease subtype. In addition to routine histology, direct immunofluorescence (IF) microscopy may demonstrate C3 and/or IgG at the dermal-epidermal junction and deposition of IgM as so-called colloid bodies (20). The overall goal of treatment is symptom control and resolution of the skin lesions. Selection of treatment should be based on the severity of the disease, the extent of the subjective symptoms, as well as taking into account relevant co-morbidities (14). Cutaneous LP is usually self-limiting and resolves within 6 months in over 50% of patients and within 18 months in up to 85% of patients (14, 21). By contrast, mucosal LP is often chronic and may be refractory to treatment (22, 23). LP with hypertrophic cutaneous lesions and isolated nail or scalp involvement is also often chronic in nature. Persistent cutaneous and mucosal lesions are considered as a premalignant condition. Thus, patients should be followed up regularly for both, adjustment of treatment, and screening for the development of malignancies.

**Table 1:** Overview of clinical subtypes and rare variants.

Cutaneous lichen planus	•Localized cutaneous lesions of LP •Generalized cutaneous LP •Hypertrophic LP •Palmoplantar LP •Atrophic LP •Actinic LP •Vesiculobullous LP •Annular LP •Erosive and ulcerative LP •Annular LP •LP pigmentosus •Lichen planus pemphigoides •Linear LP •Follicular LP	
Mucosal lichen planus	•Oral LP	° LP plaque-like or erosive ° Atrophic LP lesions of the oral mucosa ° Bullous LP of the mucosa
	•Genital LP	° Papular genital LP ° Hypertrophic genital LP ° Chronic erosive LP lesions in genitalia
	•Esophageal LP	
	•Laryngeal LP	
Appendageal lichen planus	•Lichen planopilaris (LPP)	° Classic form LPP ° Frontal fibrosing alopecia ° Graham-Little-Piccardi-Lasseur Syndrome
	•LP of the nails	
Other forms of LP	•Drug-induced lichen planus •Overlap syndromes: LP erythematosus •Lichenoid reaction of graft-vs.-host disease •Lichenoid keratosis •Ocular LP •Aural and urethral LP	

## Epidemiology

The prevalence of LP is 0.89% in the general population and 0.98% in patients seeking dermatological care according to a recent meta-analysis of 46 studies (24). The prevalence of cutaneous LP was reported to range between 0.2 and 1.0% of the adult population, and it is outnumbered by oral LP in most study populations (1, 9). The incidence of LP is less well-characterized and displays considerable geographical heterogeneity as it ranges between 14 and 250 cases/100,000 person-years (25–29). This variability more likely mirrors methodological differences in the sampled populations rather than the existence of an ethnic pre-disposition. Moreover, the aforementioned studies adopted various eligibility criteria and pooled patients with oral and cutaneous LP together. While oral LP affects females more frequently than males (24, 30), cutaneous LP does not demonstrate a prominent sex predilection (21). Cutaneous LP tends to manifest during the fifth and sixth decades of life, with almost two-thirds of patients presenting with the disease between the ages of 30 and 60 years (9, 31, 32). Oral LP tends to develop 10 years later than cutaneous LP (33). While no ethnic predilection is renowned in LP, a recent meta-analysis revealed that the pooled prevalence of oral LP was lower among patients of Asian ancestry (24). The epidemiology of LP remains to be fully delineated as the current knowledge stems mainly from scattered small-scale retrospective studies. Given that the care of patients with LP spreads across different medical specialties, in both primary and specialized healthcare, precise estimation of its incidence and prevalence is methodologically challenging.

## Pathogenesis

### Genetics

The observation of familial LP (34), the occurrence of LP in monozygotic twins (35) and HLA-based susceptibility association studies all point toward a genetic pre-disposition for LP. Several HLA alleles are associated with LP, for example between HLA-B27, HLA-B51, HLA-Bw57 (oral LP in English patients), HLA DR1 (cutaneous/oral LP), HLA-DR9 (oral LP in Japanese and Chinese patients), HLA DR6 (HCV-associated oral LP), and HLA DRB1^*^11 and DQB1^*^03 alleles (lichen planopilaris) (17, 36–40). So far, only one genome-wide association study (GWAS) has been published in LP. In total, 261 patients with hepatitis C infection with (*n* = 71) or without (*n* = 190) LP were genotyped. The findings were validated in a small group of patients (*n* = 45), of which only 7 were affected by LP. In addition to the association with the HLA, single-nucleotide polymorphisms (SNP) in loci encoding for *NRP2* and *IGFBP4* that increase or reduce risk of LP association, respectively, were found (37). Recently, a phenome-wide association study confirmed the HLA association in LP and additionally found two additional SNPs to be associated with LP. These SNP encode for three genes: *TSBP1, HCG23*, and *BTNL2* (41). Further gene associations had been described for several cytokines (IFN-γ, TNF, TNFαR, IL-4, IL-6, IL-18) and others (NFκB, PGE2, Prothrombin) (40).

### Environmental Factors

**Figure 2:**
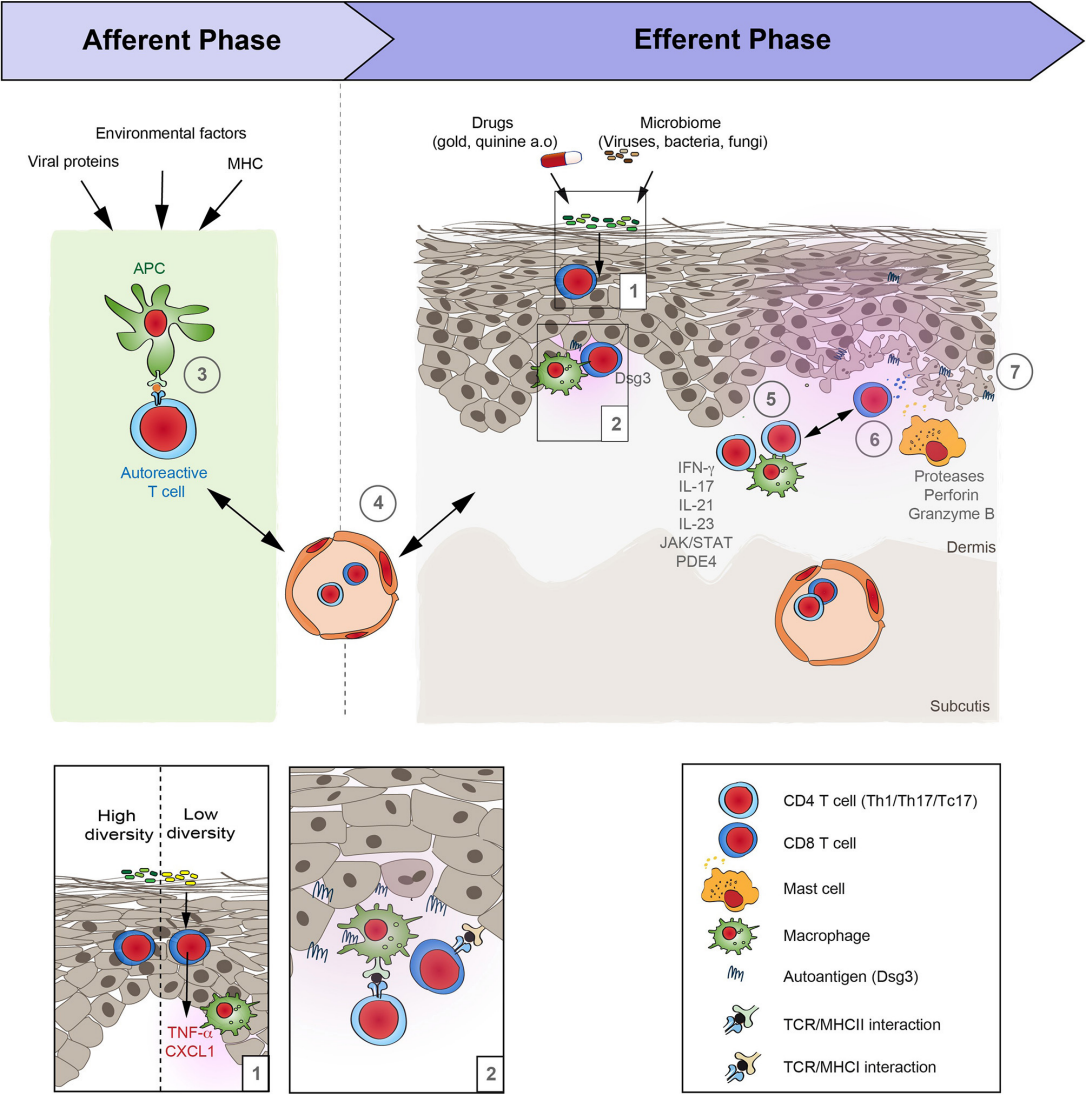
Schematic overview of lichen planus pathogenesis. Based on the increasing evidence of the autoimmune nature of lichen planus (LP), its pathogenesis may be divided in two distinct phases: Afferent phase, where tolerance to autoantigens is lost, and the efferent phase that is characterized by a T cell-driven skin inflammation. (1) Under steady state conditions the diversity of the cutaneous microbiome can alter the “inflammatory state” of the skin. If the diversity of the bacterial communities on the skin is low, this is associated with an increased expression of pro-inflammatory cytokines such as TNFα and CXCL1, as well as an increase of CD11c, suggesting an increased presence of macrophages. (2, 3) Following this presumed injury, the loss of tolerance in LP occurs in the context of an association with the MHC that may additionally be shaped by associated viral infections and other environmental factors. The site of the APC/T-cell interaction is so far unknown. It may occur locally and/or in skin draining lymph nodes. (4) Next, autoreactive T cells reach the skin by extravasation from the blood vessels. (5) Within the skin, T cells become activated by binding to the specific autoantigens. Effector functions of cytotoxic T cells is mediated by (6) proteases, such as granzyme B, as well as perforin. In addition, (7) mast cells become activated and may further aggravate inflammation in LP. However, the precise sequence of events and their interactions are only incompletely understood.

Several environmental factors have been implemented to trigger LP. Systemic viral infection, such as hepatitis C, may modify self-antigens on the surface of basal keratinocytes, or alter the immune balance, promoting a lichenoid inflammation (15–18, 42). The association between LP and hepatitis C has recently been substantiated in a large cohort study. Here, the prevalence of chronic inflammatory skin disease, including LP, was contrasted in over 23,000 patients with hepatitis C and a 3-fold greater number of non-hepatitis controls. In this study, the adjusted hazard ratio (HR) for subjects with hepatitis C to develop LP was 13.14 (95% CI: 7.10–24.31), indicating a significantly higher risk to develop LP for patients with hepatitis C. Among all evaluated chronic inflammatory skin diseases, the HR to develop LP for patients with hepatitis C was the second highest (43). Other viruses that are associated with triggering LP are members of the human herpesvirus (HHV) family, specifically (HHV)-6 and HHV-7 (44). However, studies relating to HHV-6 were not validated in other studies (45). Moreover, localized skin disease due to herpes simplex, varicella zoster, or human papilloma virus 16 (46–52) may cause LP. There are also reports that vaccine administration, including influenza and hepatitis B virus vaccines, may be associated with the development of LP (53). Additional environmental factors have been implicated in the development of oral LP. These include changes in the oral microbiome (e.g., *Candida* sp., various other bacterial infections) and dental metals precipitating allergic contact reaction (54–57). In line with this observation, the diversity of the skin bacterial communities may be involved in LP pathogenesis. Under steady-state conditions, a low diversity of bacterial communities on the skin are associated with an increased expression of proinflammatory cytokines (TNFα and CXCL1) and CD11c, pointing toward an increased infiltration with macrophages (58). These cytokines, as well as macrophages are also found in lesional LP skin (59–61). Thus, a low diversity of cutaneous bacterial communities may generate a pro-inflammatory state, even under steady-state conditions, that shares features of LP (Figure 2); thereby, potentially lowering the threshold for LP to develop. Among metals that may be associated with oral LP include amalgam (mercury), copper, and gold. Drugs may also elicit lichenoid-like reactions, which may be both clinically and histologically indistinguishable from classic LP. The most commonly implicated drugs (Table 2) are angiotensin-converting enzyme inhibitors, thiazide diuretics, antimalarials, anti-inflammatory drugs, antimicrobials, antihypertensives, psychiatric drugs, antidiabetics, PD-1-inhibitors, quinidine, penicillamine, and metals (62–65). Another peculiar potential environmental trigger for LP is UV-filters in sunscreens and hair-care products that have been noted to be associated with frontal fibrosing alopecia and lichen planopilaris (66, 67).

**Table 2:** Drugs associated with lichen planus like eruptions.

Antidiabetics			
Chlorpropamide	Glyburide	Tolazamide	Tolbutamide
Antihypertensives			
Captopril	Enalapril	Labetalol	Methyldopa
Propanolol	Diazoxide	Doxazosin	Nifedipine
Prazosin			
Antimalarials			
Chloroquine	Hydroxychloroquine	Quinacrine	
Antimicrobials			
Ethambutol	Griseofulvin	Isoniazid	Ketoconazol
Primethamine	Streptomycin	Sulfamethoxazole	Tetracyclines
Diuretics			
Chlorothiazide	Furosemid		
Hydrochlorothiazide	Spironolactone		
Metals			
Gold salts	Aresenic	Mercury	Bismuth
Palladium			
NSAIDs			
Acetylsalicylic acid	Difunisal	Fenclofenac	Flurbiprofen
Benoxaprofen	Ibuprofen	Indomethacin	Naproxen
Suldinac			
TNF-α inhibitors			
Etanercept	Infliximab	Adalimumab	Lenercept
Others			
Allopurinol	Amiphenazole	Anakinra	Cinnarizine
Cyanamide	Dapsone	Gemifrozil	Hydroyurea
Imatinib	Interferon-α	Iodides	Isotretinoin
Levamisole	Lithium	Mercapto-propiomglycine	Mesalamine
Methycran	Nivolumab	Omeprazole	Orlistat
Prembrolizumab	Penicillamine	Procainamide	Propylthiouracil
Pyrithioxin	Simvastatine	Quidine	Quinine
Rituximab	Sildenafil	Sulfasalazine	Trihexyphenidyl

## Cellular and Molecular Pathogenesis

Most of the findings on LP pathogenesis are based on morphology. Only a limited number of studies also demonstrated a functional impact of cells and/or molecules on LP pathogenesis. A cell-mediated immune response is at the core of LP pathogenesis, with cytotoxic, CD8+ T-cells in the center (Figure 2). Yet, both CD4+ and CD8+ T-cells accumulate in the dermis and oral mucosa, whilst a CD8+ T-cell-dominant infiltrate is seen within the epidermis (3, 4). Other groups have reported that CD8+ and CD45RO+ T-cells are the major cell type in the inflammatory infiltrate and that the T cell receptor (TCR) αβ, and to a lesser extent TCR γδ, are expressed (68). The functional contribution of T-cells to LP pathogenesis is further supported by a recent study that showed granule exocytosis with the release of perforin and granzyme B. In this context, to a lesser extent, the Fas/Fas-ligand system appears to be involved, the main pathway of cytotoxicity by CD4+ and CD8+ T-cells in humans (5). In addition to T-cells, mast cells may contribute to LP pathogenesis given that they are often found in the inflammatory infiltrate and show signs of activation (69–72). Immunohistochemistry of oral LP also demonstrated the presence of dendritic cells (73). The fact that CD8+ T-cells and mast cells are detected in lesions of LP patients led to the conclusion that non-specific mechanisms like mast cell degranulation and protease activation are involved in the pathogenesis of LP. These mechanisms may combine to cause T-cell accumulation in lesions and induce keratinocyte apoptosis (74). In line, an increased protease expression has been described in LP lesions that potentially contributes to the disruption of the basement membrane gelatinases (e.g., MMP-2, MMP-7, and−9), chymase, tryptase, capthepsins and caspase-3 (74–79).

Several alterations in the expression of cytokines and chemokines in lesions or serum of patients with LP have been described. Serum levels of interleukin (IL)-5, IL-6, IL-8, IL-9, IL-10, IL-12 IL-17, IL-22, tumor necrosis factor-α, transforming growth factor-β, interferon (IFN)-γ, CXCR-3, CXCR-4, CXCL-10, CXCL-12, CCR1, CCR3, CCR4, CCL5-CCR5, and CCL17-CCR4) have been found elevated (80–89). In addition, an increased expression IFN-γ and IL-17 in the skin of LP lesions has been described (81, 90)—albeit some other studies refuted these observations (91). Case reports indicated that off-label treatment of LP with Janus kinase (JAK) inhibitors (JAKi), such as tofacitinib, led to marked improvement of the disease (92–94). As IFN-γ-induced signaling centers on the activation of JAK (95), IFN-γ and JAK are likely to be central to the pathogenesis of LP. Functional evidence for a pathogenic contribution of IL-17, including the IL-17 pathway, stems from the observation of increased IL-17 and IL-23 expression in LP (84), as well as the clinical improvement following off-label treatment of LP patients with the anti-IL-17 antibody secukinumab, or the IL-12/23-targeting ustekinumab or the IL-23 inhibitor guselkumab. Of note, clinical improvement of LP following IL-17 or IL-23 blockade was accompanied by a strong reduction of the Th1 and Th17/Tc17 cellular mucosal and cutaneous infiltrates (96). This supports the previously mentioned notion that these T-cell subsets may be key effector cells in LP.

## Animal Models

The relative lack of functional insights into LP pathogenesis may be due to the limited number of pre-clinical model systems. So far, only one mouse model has been established that resembles aspects of LP pathogenesis. This model is based on the intradermal transfer of autoreactive CD4+ T-cells producing IFN-γ and TNF into syngeneic mice, inducing cellular infiltrates with epidermotropism with basal vacuolar degeneration and colloid bodies (2). Furthermore, desmoglein (Dsg) 3-specific T-cells are also capable of inducing histologically LP-like changes (97). Transfer of Dsg3-specific T-cells into immunodeficient mice induced an interface dermatitis (a distinct form of T-cell–mediated autoimmunity) in mice. The induction of the interface dermatitis depended on the specificity of the T-cell receptor as well as IFN-γ (97).

## Disease Associations

Besides systemic viral infection, several other diseases were shown to be associated with LP. A high prevalence of thyroid disease is found amongst patients with oral LP (98), whereas the association between LP and diabetes mellitus is less well-established (99). In addition to the association with chronic inflammatory diseases, patients with LP present a higher risk for dyslipidaemia, which could be explained by the cytokines involved in the pathogenesis of the disease, such as TNF-α, IL-6, IL-10, and IL-4 (100, 101). Autoimmune diseases such as alopecia areata, ulcerative colitis, vitiligo, morphea, lichen sclerosus and myasthenia gravis are over-represented in patients with LP (14).

## Diagnosis

### Clinical Manifestations

Over 20 different clinical manifestations of LP are described (Table 1). Herein, we focus on the most common variants, as well as LP pemphigoides, lichenoid GVHD and lichenoid drug eruptions.

#### Cutaneous Lichen Planus

The hallmark of cutaneous LP are purple or violet, polygonal, shiny, flat-topped, firm, papules, and plaques with white streaks (Wickham striae) (40). Wickham striae are best visualized by dermoscopy (102, 103). The cutaneous lesions may vary in size from several millimeters to more than one centimeter. The lesions may be clustered or disseminated and whilst the typical locations are the wrists, lower back, and ankles, a distribution in photo-exposed areas is also well-recognized (Figures 1a–c). Skin conditions may also appear following the lines of trauma (isomorphic response, Figure 1f). The dominant subjective symptom is pruritus, which may be severe and refractory to standard anti-pruritic therapies.

#### Mucosal Lichen Planus

The typical lesions of mucosal LP are painful and persistent erosions (erosive LP) or diffuse erythema and peeling of the mucosa (desquamative LP) (40). In addition, Wickham striae may be present in a lacy or fern-like pattern. Mucosal LP can be further subclassified into oral LP, affecting the buccal mucosa, the tongue, and to a lesser extend the gums and lips (Figures 1m,n) or genital LP, affecting the glans penis, labia majora, labia minora and vaginal introitus (Figures 1p–r). Chronic disease may result in scarring, with the formation of adhesions, resorption of labia minora and ultimately introital stenosis. Penile LP usually presents with papules around the glans penis, white streaks and erosions. In rare cases, mucosal LP may also affect the lacrimal glands, eyelids, external ear canal, esophagus, larynx, bladder, and anus.

#### Lichen Planopilaris

Lichen planopilaris (LPP) presents as tiny red spiny follicular papules and extending smooth areas on the scalp or less often, elsewhere on the hair-bearing regions body areas (104, 105). Destruction of the hair follicles leads to permanently bald patches characterized by sparse “lonely hairs” (Figures 1i,j). Frontal fibrosing alopecia is a variant of LPP that affects the anterior scalp, forehead and eyebrows. Another subtype of LPP is Graham-Little-Piccardi-Lasseur Syndrome with the following characteristics: multifocal, patchy, cicatricial alopecia present on the scalp, non-cicatricial alopecia of the axillae, non-cicatricial alopecia of the perineum, and follicular hyperkeratosis of the trunk and extremities (106).

#### Nail Lichen Planus

LP may affect one or more nails (Figure 1l), sometimes in the absence of skin involvement. LP thins the nail plate, which may become grooved and ridged. The nail may darken, thicken or lift off the nail bed (onycholysis). Sometimes, the cuticle is destroyed and forms a scar (pterygium). The nails may shed or stop growing altogether, and they may rarely, completely disappear (anonychia). An important clinical feature of nail LP is the occurrence of a dorsal pterygium.

#### Lichen Planus Pemphigoides

LP pemphigoides is clinically characterized by the simultaneous occurrence of lichenoid and bullous skin lesions. By some, LP pemphigoides is considered as an autoimmune dermatosis with autoimmunity toward type XVII collagen (COL17). By contrast, others consider LP pemphigoides the co-occurrence of 2 independent skin diseases, or as a variant of LP (14, 107, 108).

#### Lichenoid Graft-vs.-Host-Disease

Graft-vs.-host disease (GVHD) is the primary complication of allogeneic bone marrow transplantation and the skin is the most commonly involved organ. The clinical picture varies and often is similar to autoimmune or inflammatory diseases. Cutaneous GVDH can imitate classical lichen planus with purple, polygonal, pruritic papules (but without Wickham striae) or lichen planus pigmentosus (109, 110). GVHD refers to the inflammatory manifestations, when immunocompetent T-cells from a donor recognize and react against “foreign” tissue antigens in an immunocompromised host, this autoreactive pre-condition leads to a Th2 immune response induced interface dermatitis (109).

#### Lichenoid Drug Eruptions

Lichenoid drug eruptions often mimic idiopathic lichen planus although there can be features that may help to distinguish them, which may include: symmetrical rash on the trunk and limbs, predominantly in sun-exposed areas. Skin features do normally not show Wickham striae, nail and mucous membrane involvement is missing. Medications reported to trigger a lichenoid drug eruptions are, exemplary (14): ACE inhibitors, beta-blockers, nifedipine, methyldopa, hydrochlorothiazide, frusemide, spironolactone, non-steroidal anti-inflammatory drugs (NSAIDs), carbamazepine, phenytoin, ketoconazole, 5-fluorouracil, imatinib, hydroxychloroquine, sulfonylurea, dapsone, mesalazine, sulfasalazine, allopurinol, iodides and radiocontrast media, interferon-α, omeprazole, penicillamine, tetracycline, infliximab, etanercept, adalimumab, imatinib, misoprostol, sildenafil, and herpes zoster/influenza vaccines.

Contact allergies also may mimic lichen planus: Oral lichenoid lesions may be associated to type-IV-sensitization to mercury or dental amalgam (111, 112); lichenoid skin lesions usually result from contact with rubber, chemicals used in clothing dyes or chemicals in wine industries (113).

### Confirmation of Diagnosis

#### Histopathology

A skin/mucosal biopsy is recommended to confirm the diagnosis of LP. The typical histological findings are acanthosis and hyperkeratosis, wedge-shaped hypergranulosis, vacuolic degeneration of the basal layer, alteration or loss of rete ridges resulting in a sawtooth appearance and a dense, band-like lymphocytic infiltrate in the upper dermis along the dermal-epidermal junction (Figures 1h,k,o,s). Apoptotic keratinocytes are often seen near the basal layer and are termed colloid bodies. For LP affecting the scalp, for example LPP, shows beside the penitent LP features often the destruction of hair follicle root sheaths and follicular plugging as well as the loss of sebaceous glands as well (114) (Figure 1k).

#### Immunofluorescence

Additionally, a lesional biopsy for direct IF microscopy can be a useful, especially when trying to differentiate between LP and other autoimmune diseases, such as pemphigus vulgaris, mucous membrane pemphigoid, or lupus erythematosus (LE) (115, 116). In LP, direct IF microscopy (Figure 1g) may reveal globular deposits of IgA, IgM, IgG, C3, or fibrinogen mixed with apoptotic keratinocytes (117, 118).

## Differential Diagnoses of LP

### Cutaneous Lichen Planus

The differential diagnosis of cutaneous LP is broad and includes graft-vs. host-disease, psoriasis vulgaris, guttate psoriasis, secondary syphilis, pityriasis lichenoides, pityriasis rosea, lichen nitidus, lichen simplex chronicus, lichen sclerosus, lichen striatus, linear epidermal naevus, eczema, prurigo nodularis, erythema dyschromicum perstans, eczematid-like purpura, drug eruption, granuloma annulare, lichen amyloidosus, Kaposi sarcoma and lupus erythematosus. In most cases, histology permits a reasonable differentiation between these diseases and inflammatory disorders.

### Mucosal Lichen Planus

Similarly, an extensive list of differential diagnoses should be considered when diagnosing LP of the oral cavity including pemphigus vulgaris, mucous membrane pemphigoid, lupus erythematosus, secondary syphilis, traumatic patches, and candidiasis. Vulval/penile LP can be difficult to distinguish from lichen sclerosis, mucous membrane pemphigoid, psoriasis, intraepithelial neoplasia, graft-vs.-host disease, erosive dermatitis, and intertrigo. Histology and direct IF microscopy should allow a definite diagnosis of LP and the exclusion of other diseases.

### Lichen Planopilaris

Various diseases may appear similar to LPP, especially when the destruction of the hair follicles leads to permanently bald or even scarring patches without inflammation or tiny red spiny follicular papules, such as patchy alopecia in systemic LE, alopecia areata, diffuse alopecia due to secondary syphilis or severe folliculitis. Brunsting-Perry cicatricial pemphigoid is rare variant of mucous membrane pemphigoid associated with scarring on the head and neck region. Differentiating them can be difficult, besides punch biopsy trichometric analysis, fungal culture, blood tests are recommended to find the underlying medical condition.

### Nail Lichen Planus

Nail LP can be challenging to differentiate from psoriasis, atopical dermatitis, alopecia areata and onychomycosis. For the later, appropriate laboratory testing for presence of fungi is recommended.

## Management

The ultimate aim of treatment is the resolution of the skin lesions and their associated symptoms. This is particularly important in oral LP where painful erosions can result in significant malnutrition and weight loss. Drug-induced LP should always be considered and excluded prior to commencing immunosuppressive therapy (14) and the responsible drug discontinued or substituted. An LP-associated diseases should be checked in each patient. Hypertrophic and mucosal LP lesions are potentially premalignant and regular follow-up and biopsies should be considered to exclude malignant transformation (Table 3).

**Table 3:** Management of lichen planus.

	**First-line**	**Second-line**	**Third-line**
Cutaneous lichen planus (LP)	•Topical steroids •Intralesional steroids •Systemic corticosteroids •Acitretin/isotretinoin •Cyclosporine	•Topical calcineurin inhibitors •Phototherapy (UVB or PUVA) •Combination of phototherapy and acitretin •Sulphasalazine	•Hydroxychloroquine •Azathioprine •Mycophenolate mofetile •Methotrexate •Apremilast •Ustekinumab •Topical calcipotriol •Antibiotic treatment (trimethoprim–sulphomethoxazole, metronidazole) •Antifungal therapy (traconazole, terbinafin, griseofulvin) •Cyclophosphamide •Thalidomide •Adalimumab •Interferon a2b •Alitretinoin •Low molecular weight heparin •Photodynamic therapy •Extracorporeal photochemotherapy •Laser
Mucosal LP	•Topical steroids •Intralesional steroids •Systemic corticosteroids •Acitretin/isotretinoin •Topical retinoids •Cyclosporine	•Topical calcineurin inhibitors •Hydroxychloroquine •Azathioprine •Sulphasalazine •Mycophenolate mofetile •Methotrexate •Adalimumab •Etanercept	•Cyclophosphamide •Thalidomide •Antibiotic treatment (metronidazole, trimethoprim–Sulphomethoxazole, tetracycline, doxycycline) •Antifungal therapy (traconazole, griseofulvin) •Dapsone •Low molecular weight heparin •Interferon •Topical tocopherol •Photodynamic therapy •Extracorporeal photochemotherapy •Laser

*Treatment recommendations shown in the table are based on the European S1 guidelines (14)*.

### Cutaneous Lichen Planus

The first-line treatments for limited LP are (super)potent topical steroids, with intralesional steroid injection reserved for hypertrophic and/or unresponsive lesions (14, 119, 120). For disseminated disease, systemic corticosteroids can be considered, either as oral therapy or intravenous “pulse” therapy, to achieve disease control. Thereafter, the oral dose can be tapered or the interval between intravenous administrations extended (14, 121, 122). Other first-line therapies include systemic retinoids (acitretin/isotretinoin) or cyclosporine (14, 123–126). If diffuse cutaneous LP remains unresponsive, second-line therapy should be considered. These include sulphasalazine, and phototherapy such as broadband/narrowband UVB or psoralen and UVA (PUVA), and the combination of UV/PUVA with retinoids (14, 121, 127–130). For topical treatment of limited and diffuse cutaneous LP calcineurin inhibitors can be used to reduce side-effects of topical steroids (14, 131). Third-line treatments include hydroxychloroquine, azathioprine, methotrexate, mycophenolate mofetil, or biologics targeting IL-12/23 (14, 121, 130, 132–136). Based on the fact that in LP proinflammatory signaling pathways result in T-cell-dependent immune response, oral JAKi may represent a future treatment option (137). Oral antihistamines may be helpful to minimize the itch (14). Topical antipruritic agents such as menthol, camphor, or polidocanol can be prescribed as an adjuvant to the main treatment (14). The majority of patients with cutaneous lesions spontaneously clear within 12–24 months (21); however, relapses are common. Healing may also be complicated by the development of post-inflammatory hyperpigmentation (1, 9).

### Mucosal Lichen Planus

Mucosal LP is often difficult to treat, particularly when extensive erosions are present. Long-term follow-up is necessary to monitor disease activity and to exclude malignant transformation of erosive lesions (14). The mainstay of treatment of mucosal LP are topical corticosteroids (14, 138). Superpotent steroids can be applied topically (in the form of an adhesive paste) twice daily for 1–2 months, and then administered as required (14). Intralesional steroid injections are worth considering when lesions are particularly painful and fail to respond to topical therapy (14, 138, 139). Systemic corticosteroids are reserved for patients with severe erosive mucosal LP (recalcitrant, multi-site, ulcers) and to more rapidly induce a remission (14). Further systemic first-line treatments are retinoids (acitretin/isotretinoin) and cyclosporine (14). Second-line treatments include sulphasalasine, azathioprine, hydroxychloroquine, methotrexate, mycophenolate mofetil, and/or use of topical calcineurin inhibitors (14, 133, 140–147). Third-line treatment may include cyclophosphamide, thalidomide, metronidazole, trimethoprim–sulphomethoxazole, antibiotic treatment, itraconazole, griseofulvin, dapsone and extracorporeal photochemotherapy (ECP) (14, 148–156).

In the management of **oral LP**, lidocain solution as mouthwash may be helpful to reduce pain. Amphotericin B solution as mouthwash several times daily (after food consumption) may prevent secondary candida infection. Patch tests may be recommended for patients with oral lichen planus affecting the gums and who have fillings with amalgam, to assess for contact allergy to thiomersal, a mercurial compound (14). Mucosal LP may clear spontaneously within 5 years, but typically it is a chronic disease with a remitting and relapsing course (22, 23).

The general principles of the management of **genital LP** are similar to those of LP confined to the oral mucosa (14). Most cases of papulosquamous genital LP are self-limited, and treatment with emollients and mid-potency steroids for a few weeks leads to complete remission. First-line treatment for erosive LP of the vulval or penile mucosa are superpotent topical corticosteroids (14, 157), which can be gradually tapered (14). Calcineurin inhibitors (tacrolimus/pimecrolimus) are a further topical treatment option (14). The aim for the treatment of erosive genital lesions is the prevention or limitation of scarring. In women, synechia formation with vaginal stenosis may be prevented by the use of vaginal dilators and application of intra-vaginal steroids to treat mucosal inflammation (14). In uncircumcised men, circumcision is usually recommended to avoid phimosis (14). Local anesthetic gel, low-dose tricyclic antidepressants or anticonvulsants may relieve itch and ease discomfort and nystatin cream can prevent secondary fungal infections (14).

### Lichen Planopilaris

The aim of the treatment is disease control to prevent permanent hair loss due to scarring (14). Furthermore, treatment can reduce itching and burning of the scalp. Topical steroids are treatment of first choice (97, 158–160). Intralesional steroid injections may improve response rates (161). Topical calcineurin inhibitors may be used as monotherapy or as an adjuvant to systemic therapy proved effective (162). Systemic steroids are the mainstay of treatment for rapidly progressive disease to prevent scarring (163), while introducing cyclosporine, methotrexate, or hydroxychloroquine as steroid sparing agents (160, 164–168). Suggested second-line options are retinoids (acitretin/isotretinoin), tetracycline/doxycycline, mycophenolate mofetil, adalimumab, pioglitazone, thalidomide, or rituximab (160, 162, 165, 167, 169–175).

### Nail Lichen Planus

LP of the nails is generally difficult to treat and the prognosis is poor (14). LP affecting the nails frequently leads to permanent destruction of the nail matrix and bed with functional limitations. Therefore, early treatment is essential, even in mild cases of nail LP (176). Potent topical steroids under occlusive dressings are the preferred, first-line topical treatment (14). Due to the poor short-term efficacy of topical steroids and long-term side effects, triamcinolone acetonide injections (intralesional) should be considered as further first-line therapies (176, 177). Oral prednisone 0.5 mg/kg for 3 weeks demonstrated a marked improvement and is useful when multiple nails are affected (14). Oral retinoids are second-line choices (178–180), and immunosuppressive agents may also be considered (14, 181, 182). In a case series, topical tacrolimus ointment 0.1% was successfully used in treatment of nail LP (183).

#### Lichenoid Graft-vs.-Host-Disease

Corticosteroids are the backbone the treatment of cutaneous lichenoid GVHD, but ~30% need additional immunosuppressant such as cyclosporine. cyclophosphamide, methotrexate, azathioprine, mycophenolate mofetil, pentostatin, or high-dose thalidomide and hydroxychloroquine (184). Another option for skin involvement might be phototherapy, while extracorporeal photochemotherapy may improve cutaneous as well as systemic involvement (184).

#### Lichenoid Drug Eruptions

The triggering agent should be stopped (14); improvement of the skin lesions can take weeks to months. Commonly flat pigmented freckles persist and fade more slowly. Steroids (topical/systemically) may be supportive to give relief or rapid resolution.

#### Emerging Treatments

In LP, new therapeutic options currently stem from case reports and/or case report series. These have set the rationale for the planning of current clinical trials in LP (Table 4). The molecular targets currently persued for LP can be categorized into biologics targeting cytokines and small molecules blocking intracellular signaling. In addition, photodynamic therapy has consistently been reported to have favorable outcomes in LP patients (185).

**Table 4:** Selected clinical studies in lichen planus.

**Study**	**Lichen planus**	**Intervention**	**Target**	**Design**	**Phase**	**Status**
NCT03697460	Cutaneous LP	INCB018424 (Ruxolitinib)	JAK1/2	Single center, exploratory, open-label, single-arm	2	Completed (2021)
NCT03656666	Genital erosive LP	Apremilast	PDE4	Double-blinded, randomized, placebo-controlled	2	Recruiting (2021)
NCT05030415	Lichen planopilaris and LP	Ixekizumab	IL17A	Open-label		Recruiting (2021)
NCT04300296	Lichen planopilaris, oral and cutaneous LP	Secukinumab	IL17A	Multicenter, randomized, double-blind, placebo-controlled	2	Active, not recruiting (2021)
NCT03417141	Lichen planopilaris	Mechlorethamin (Valchlor)	–	Open-label	2	Completed (2021)
NCT04409041	Lichen planopilaris and frontal fibrosing alopecia	Naltrexone	Opiate-receptor	Open-label	2	Completed (2021)
NCT03858634	Pruritus, CIU, LP, Lichen simplex chronicus, plaque psoriasis	Vixarelimab (KPL-716)	Oncostatin M receptor beta	Quadruple -blinded, randomized	2	Completed (2020)
NCT04976673	Oral LP	PDT	–	Double-blinded, randomized	2	Completed (2021)
NCT01282515	Female genital erosive LP	PDT	–	Single (investigator)-blinded, randomized	2/3	Completed (2021)
NCT04991012	Oral LP	PDT	–	Double-blinded, randomized	2	Completed (2021)

*A search on clinicaltrials.gov was conducted on September 25th, 2021 with the key word “lichen planus”. A total of 116 clinical studies were identified. We focused on studies with a molecular target. A total of 14 completed interventional studies with molecular targets were updated since 2020. In addition, 2 studies with a defined molecular target (status: not recruiting/recruiting, enrolling by invitation/active, not recruiting) were found. We also included completed (with updates since 2020) and ongoing studies using photodynamic therapy. Number in brackets in the Status column indicate the last year, the study was updated in clincaltrials.gov. CIU, Chronic idiopathic urticarial; LP, Lichen planus; PDT, Photodynamic therapy*.

Currently licensed (for other indications than LP) biologics targeting IL-17 or the IL-17R are secukinumab, ixekizumab and brodalumab. In 2017, occurrence of oral LP was noted in a psoriasis patient treated with secukinumab. As concurrently oral candidiasis, a relatively common adverse event under anti-IL-17 treatment, was present, the causality of IL-17 inhibition and induction of oral LP remained ambiguous (186). In addition to this case, 3 more cases of cutaneous/oral lichenoid eruptions associated with IL-17 inhibition were noted, albeit (oral) LP was not formally diagnosed (187–189). By contrast, response to IL-17 inhibition has been reported in a total of 5 LP patients (96, 190, 191). Grounded on the latter observations, as well as the increased serum and tissue IL-17 expression in both oral and cutaneous LP (83, 192, 193), two clinical studies currently evaluate the impact of IL-17 inhibition using secukinumab or ixekizumab in patients with LP (NCT04300296, NCT05030415).

Biologics targeting either IL-12 and/or IL-23 (ustekinumab) or IL-23 alone (risankizumab, tildrakizumab and guselkumab) have so far not been associated with the induction of LP. However, one report noted a failure of LP to respond to ustekinumab (194). Later observations noted a response of IL-12/23 inhibition on one patient with LP pemphigoides (195), and three patients with LP (96, 196). Use of IL-12/23 was grounded on the observation of increased IL-23 expression in oral LP (84), as well as an increased serum concentration of IL-23 in LP patients (197). Of note, we are not aware of any study addressing the impact of IL-12/23 inhibition in LP.

By contrast to IL-17 and IL-23, blockade of TNF-α has not emerged as a promising therapeutic target in LP. There are several reports on lichenoid drug eruptions following TNF-α inhibition (198, 199), and only one report on a successful treatment of lichen planus with the anti-TNF-α antibody adalimumab (135). On the expression level, increased TNF-α expression has been noted in the skin and serum of LP patients (60, 90). In line, a study evaluating the impact of the TNF-α inhibitor etanercept of LP was terminated due to slow recruitment in 2018 (NCT00285779).

Recent work showed that the inflammation in LP is dominated by an IFN-γ and an IL-21 signature, along with an increased expression of phospho-STAT1 in the dermal infiltrate (200). In another T-cell mediated inflammatory skin disease, namely alopecia areata, the identification of an IFN gene signature in affected skin identified JAK inhibitors as potential new treatments for alopecia areata, which showed efficacy in phase 2 clinical trials (201, 202). Based on these morphological observations and considerations, the authors concluded that use of JAK inhibitors may be beneficial in LP (200). Successful treatment of a treatment-refractory LP patient with the JAK1/3-selective JAKi tofacitinib supports this notion. Furthermore, in 2 independent case series, tofacitinib used as either monotherapy or adjunctive therapy led to clinical improvement in 11/13 patients (95, 96). In line with these observations, a clinical trial currently investigated the impact of topical ruxolitinib in LP patients. The trial was completed in 2020. Results are shown at clinicaltrial.gov: 12 patients were enrolled, 3 were lost to follow-up, most likely related to the Covid-19 pandemic, and no serious adverse events occurred (NCT03697460).

Grounded on the broad anti-inflammatory activity of the PDE4 inhibitor apremilast, the safety and efficacy of the drug in LP patient's refractory to topical corticosteroid treatment was evaluated in an investigator-initiated, single-center, non-randomized, open-label, pilot study in 2013. Patients were treated with 2 × 20 mg apremilast per day for 12 weeks. The primary endpoint was achieving a 2-grade or more in Physician Global Assessment (PGA) at 12 weeks. While all patients demonstrated a significant clinical improvement, 3/10 met the primary endpoint (137). Subsequently, a total of 5 LP patients, mostly with treatment-refractory disease, were reported to improve when treated with apremilast (203–205). Based on these reports, apremilast is currently evaluated in a randomized placebo-controlled clinical trial in women with genital erosive lichen planus (206). Currently, patients are recruited to this study (NCT03656666).

Other clinical trials are evaluating the impact of topical mechlorethamine, a topical chemotherapy used for the treatment of cutaneous T cell lymphoma (207), in LP. The study was completed in 2019, but so far results have not been published (NCT03417141). A study treating LP patients with the opiate opioid receptor antagonist naltrexone was recently completed (NCT04409041). Again, results are pending to be published. In this line, a small case series reported on the beneficial outcome of naltrexone in LPP (208).

Most of non-pharmacological interventions for LP that are currently evaluated in clinical trails focus on the use of photodynamic therapy (PDT) (NCT04976673, NCT01282515). Both studies are completed, while the results have not been published so far. Up to date, a total of 5 controlled studies has addressed the impact of PDT in oral LP (209–213). In most studies, topical steroids were used as an active comparator. In 3/5 studies, no difference between the 2 treatment modalities (that both led to a reduced severity of LP) was observed (209, 211, 212), whilst in 2/5 studies, a superior effect of PDT was noted (211, 213). In an open study using PDT in oral LP, a significant change of molecular disease markers (reduced numbers of CD4+ and CD8+ T-cells in the lesions, reduced numbers of activated T cells in the circulation) were observed in parallel to the clinical improvement (214).

In addition, a recent retrospective investigation and review of the impact of narrowband UVB phototherapy and psoralen plus UVA (PUVA) photochemotherapy as second-line treatment of LP showed a relatively good response (complete responses in a little over 70% for both narrow-bad UVB and PUVA), whereby adverse events were only observed in patients treated with oral PUVA (215).

Previous reports also indicated a good response of recalcitrant LP to extracorporeal photochemotherapy (ECP) (155, 216, 217). In a larger case series, 9/12 patients showed complete remission and 3/12 a partial response. In follow-up, relapse occurred frequently when ECP sessions were less frequent or stopped (216). Since 2010, no more reports on the use of ECP in LP were published. Hence, ECP may be used in LP cases refractory of several previous therapies, and additional treatments should be administrated to maintain the (presumed) initial good response to ECP.

## Outlook

Overall, LP is an under-recognized dermatosis, whose epidemiology and pathogenesis is only partially understood, the disease is associated with significant morbidity, and current treatment options are limited in their success. Given the lack of double-blind randomized control trials, treatment is often based on clinical experience and the results of retrospective meta-analyses (121, 218). Biological treatments (93) and JAKi (96) hold significant promise as future therapeutic options. The lack of animal models underscores the importance of a comprehensive understanding of the pathogenesis of LP elucidating human phenotype-genotype correlations facilitating renewed efforts to unravel the cellular and molecular changes underlying the disease (219). Still, with the emergence of biological treatment options and of JAKi that both derived from careful clinical observations, the treatment landscape of LP will hopefully improve in the near future.

## Author Contributions

KBo, EL, KK, RL, and KBi wrote the manuscript. All authors read, commented, and approved the final version of the manuscript.

## Funding

This work was supported by structural funding from the Deutsche Forschungsgemeinschaft through Excellence Cluster 2167/1 Precision Medicine in Chronic Inflammation and the Schleswig-Holstein Excellence Chair Program from the State of Schleswig-Holstein. KK received support from the Alexander von Humboldt Foundation (Humboldt Research Fellowship for Post-doctoral Researchers).

## Conflict of Interest

The authors declare that the research was conducted in the absence of any commercial or financial relationships that could be construed as a potential conflict of interest.

## Publisher's Note

All claims expressed in this article are solely those of the authors and do not necessarily represent those of their affiliated organizations, or those of the publisher, the editors and the reviewers. Any product that may be evaluated in this article, or claim that may be made by its manufacturer, is not guaranteed or endorsed by the publisher.
